# Supplementary material for: Bulk evidence of anisotropic s-wave pairing with no sign change in the kagome superconductor CsV3Sb5
Source: Nat Commun. 2023 Feb 7;14:667. doi: 10.1038/s41467-023-36273-x (PMC9905511; doi:10.1038/s41467-023-36273-x)
Supplement: Supplementary file 1 — Supplementary Information [file 41467_2023_36273_MOESM1_ESM.pdf]

Supplementary Information for

Bulk evidence of anisotropic s-wave pairing with no sign change in the kagome superconductor CsV_3Sb_5

M. Roppongi, K. Ishihara, Y. Tanaka, K. Ogawa, K. Okada, S. Liu, K. Mukasa, Y. Mizukami, Y. Uwatoko, R. Grasset, M. Konczykowski, B. R. Ortiz, S. D. Wilson, K. Hashimoto*, T. Shibauchi*

*Corresponding authors.

Email: k.hashimoto@edu.k.u-tokyo.ac.jp; shibauchi@k.u-tokyo.ac.jp

This PDF file includes:

Supplementary Text

Figs. S1 to S7

Tables S1 and S2

References

I. ELECTRON SCATTERING CROSS-SECTION

We calculated the cross-sections σ for collisions of high-energy electrons as a function of incident electron energy (see Fig. S1) and estimated the formation of Frenkel pairs on the Cs, Sb, and V sites. This calculation was performed using the SECTE software developed at Laboratoire des Solides Irradiés [S1]. We assumed a value of the energy for ion displacement of $E_d = 25\,\mathrm{eV}$. This is a typical value for intermetallic compounds, usually in the range of tens of eV. The exact value is not essential for our estimate as it does not change the cross-section values dramatically.

The concentration of created vacancies at each atom, n_i (i = Cs, Sb, and V), can then be evaluated through the following relation:

$$n_i = \sigma_i \phi N_i / V, \tag{1}$$

where σ_i is the electron scattering cross-section of each atom i, ϕ is the irradiation fluence (number of high-energy electrons impinging on a unit area of the sample), N_i is the number of each atom per unit cell, and V is the volume of the unit cell. With a unit cell volume of $243.3\,\mathrm{\AA}^3$ [S2], we estimate that the amount of created defects is $n_{\mathrm{Cs}} = 4.5 \times 10^{18}\,\mathrm{cm}^{-3}$,

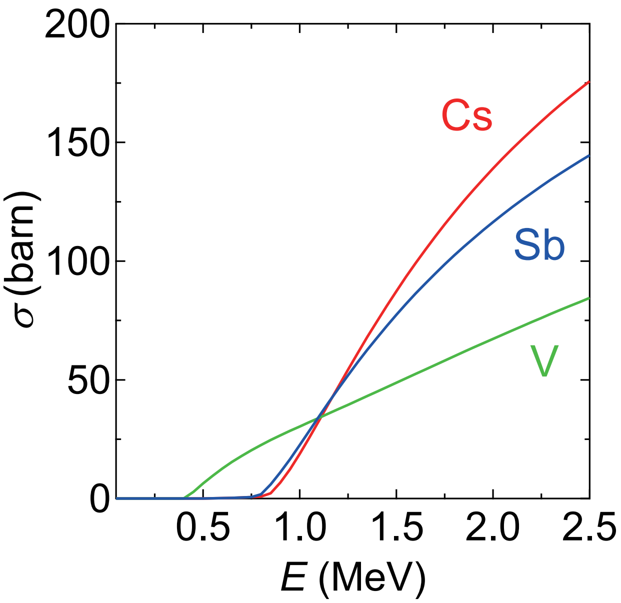

Fig. S1. **Electron scattering cross-section.** Calculated cross-sections σ for Frenkel-pair production at the Cs (red), Sb (blue), and V (green) sites of CsV_3Sb_5 as a function of incident electron energy.

$n_{\mathrm{V}} = 6.5 \times 10^{18}\,\mathrm{cm}^{-3}$, and $n_{\mathrm{Sb}} = 1.8 \times 10^{19}\,\mathrm{cm}^{-3}$ per $1\,\mathrm{C/cm}^2$ of $2.5\,\mathrm{MeV}$ electron irradiation. This estimation gives an upper limit to the concentration of defects created. Note that this calculation does not take into account partial annealing of the vacancies upon warming to room temperature after the irradiation at $20\,\mathrm{K}$, which further reduces the amount of defects.

II. ELECTRON IRRADIATION EFFECTS ON LATTICE CONSTANTS AND CARRIER DENSITY

Figure S2 shows the lattice constants of the pristine and irradiated CsV_3Sb_5 single crystals as a function of dose obtained from X-ray diffraction measurements. Although a slight sample dependence has been observed in the a- and c-axis lengths, there is no trend of increase or decrease with dose, indicating that point defects (Frenkel pairs) introduced by electron irradiation do not change the lattice constants.

Figure S3 shows the Hall resistivity as a function of magnetic field for the 8.6 C/cm^2 irradiated samples. The Hall resistivity for pristine samples in previous reports [S3, S4] shows a large temperature dependence below 50 K, accompanied by a sign reversal from negative to positive at around 30 K. Such a large temperature dependence of the Hall resistivity reflects the multiband nature of CsV_3Sb_5, indicating that the mobility of hole carriers increases significantly as lowering the temperature. Note that anomalies observed in the low-field region ($-1 \lesssim \mu_0 H \lesssim 1$) at low temperatures are discussed in terms of the anomalous Hall effect [S5]. Although one cannot accurately obtain carrier densities from the slope of the Hall resistivity in a multiband system, we simply calculate the number of hole carriers in

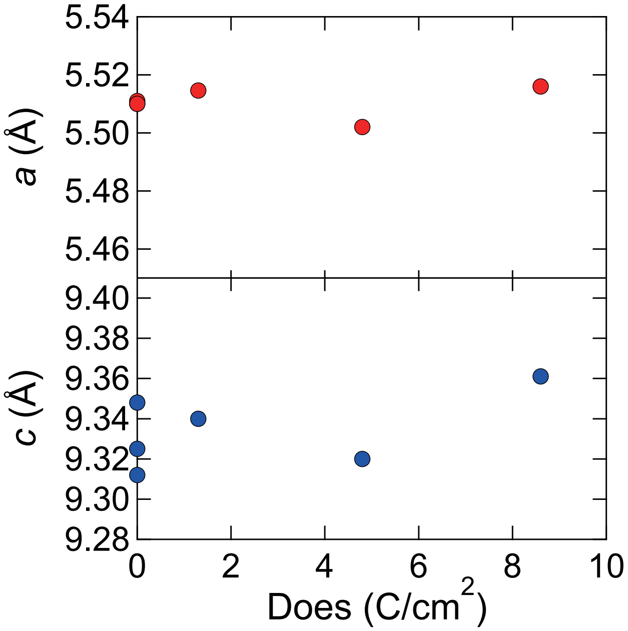

Fig. S2. **Lattice constants vs irradiation dose.** Lattice constants of the a- (upper panel) and c-axes (lower panel) as a function of irradiation dose.

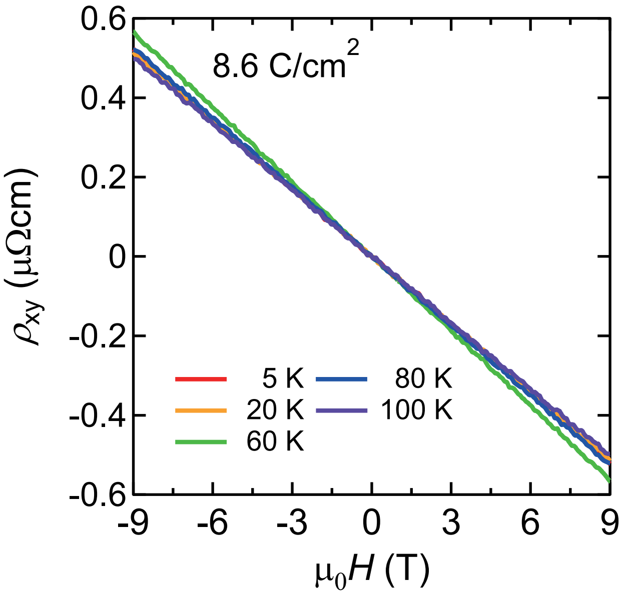

Fig. S3. Hall effect in the 8.6 C/cm^2 irradiated CsV_3Sb_5 single crystal. Hall resistivity as a function of magnetic field measured at several temperatures for the 8.6 C/cm^2 irradiated sample.

the single-band model using the Hall resistivity data above 60 K, which is estimated to be 0.7–1.0 $\times$ 10^{22} cm^{-3} [S3, S4]. This value is in the same order of magnitude as the value $\sim 1 \times 10^{22}$ cm^{-3} obtained from quantum oscillation measurements and first-principle band calculations [S6]. In contrast to the pristine sample, the Hall resistivity of the 8.6 C/cm^2 irradiated sample shows a negligibly small temperature dependence (Fig. S3), suggesting that the increase in mobility of hole carriers observed in the pristine sample was suppressed by electron irradiation. However, the slope of the Hall resistivity is almost the same as that of the pristine samples in the high-temperature region, which corresponds to 0.85×10^{22} cm^{-3} in the single-band model, indicating that the carrier density does not change with electron irradiation.

III. DEVIATION FROM MATTHIESSEN'S RULE IN ELECTRON-IRRADIATED SAMPLES

The resistivity curves $\rho(T)$ of the electron-irradiated samples do not shift parallel to that of the pristine sample, showing a deviation from Matthiessen's rule (see Fig. 1d). This can be naturally understood by considering that CsV_3Sb_5 is a multiband system. Here, for simplicity, we consider the two-carrier model with electron and hole carriers and assume that the scattering time $\tau_i(T)$ ($i = \text{electron}, \text{hole}$) is composed of two terms: one is the temperature-dependent term $\tau_{i,0}(T)$ due to electron-lattice/electron-electron scattering, and the other is the temperature-independent term τ_{imp} due to impurity scattering as follows,

$$1/\tau_i(T) = 1/\tau_{i,0}(T) + 1/\tau_{\text{imp}}. \tag{2}$$

The conductivity $\sigma(T)$ can then be given as follows:

$$1/\rho(T) = \sigma(T) = \sigma_{\text{electron}}(T) + \sigma_{\text{hole}}(T) = \sum_{i\,=\,\text{electron, hole}} \frac{n_i e^2 \tau_i(T)}{m_i^*}, \tag{3}$$

where n_i is the carrier density, m_i^* is the effective mass, and e is the elementary charge. In the single-band case, $\rho(T) = 1/\sigma(T) = \frac{m^*}{ne^2}(\frac{1}{\tau(T)} + \frac{1}{\tau_{\text{imp}}})$, which immediately leads to a parallel shift of resistivity by the temperature-independent impurity scattering, and thus Matthiessen's rule holds. However, this is not the case in the multiband system. Thus, the deviation from Matthiessen's rule we have observed in CsV_3Sb_5 can be attributed to the multiband nature of the present system.

IV. ESTIMATION OF ABSOLUTE VALUE OF PENETRATION DEPTH AT 0 K AND PAIR BREAKING PARAMETER

In general, the magnetic penetration depth at 0 K of a superconductor with disorder, $\lambda(0)$, can be described by the following equation:

$$\lambda(0) = \lambda_{\rm L}(0)(1+\xi/l)^{1/2}, \quad (4)$$

where $\lambda_{\rm L}(0)$, l, and ξ are the London penetration depth at 0 K, mean free path, and coherence length, respectively. The ratio ξ/l can be given by $\hbar/(\pi\tau\Delta(0))$ using two relations, $\xi = \hbar v_{\rm F}/(\pi\Delta(0))$ and $l = v_{\rm F}\tau$, where $\hbar$ is the Dirac constant, $v_{\rm F}$ is the Fermi velocity, $\Delta(0)$ is the superconducting gap at 0 K, and τ is the scattering time.

In the two-gap model, $\lambda(0)$ can be given by

$$1/\lambda^2(0) = 1/\lambda_1^2(0) + 1/\lambda_2^2(0), \quad (5)$$

where $\lambda_i(0)$ $(i = 1, 2)$ is the penetration depth at the i-th band. As discussed in the next section, as the gap values on the two bands, $\Delta_1(0)$ and $\Delta_2(0)$, obtained from the fitting analysis of the superfluid density are almost the same, we use the same gap value $\Delta(0) = (\Delta_1(0) + \Delta_2(0))/2$ for the two bands. We also assume that $\Delta(0)$ is independent of irradiation dose. Indeed, the obtained gap values from the fitting of the superfluid density for the irradiated samples do not significantly change with irradiation dose. In addition, the Hall resistivity data in the 8.6 C/cm^2 irradiated sample show little temperature dependence (Fig. S3), indicating that the scattering times of the electron and hole carriers are dominated by the temperature-independent impurity scattering $\tau_{\rm imp}$. Therefore, we can use the same value of τ for the two bands, which can be estimated from the relation $\tau = \mu_0\lambda_{\rm L}^2(0)/\rho_0$,

TABLE S1. **Physical parameters of pristine and irradiated CsV_3Sb_5 single crystals.**

Dose (C/cm^2)	RRR	ρ_0($\mu\Omega$cm)	τ(s)	ξ/l	$\lambda(0)$(nm)	g
pristine	84	0.403	4.67×10^{-11}	0.0136	387	0.0565
1.3 (run#1)	8.9	5.90	3.19×10^{-12}	0.199	424	0.826
3.3 (run#1)	4.7	25.2	7.47×10^{-13}	0.849	526	3.53
8.6 (run#1)	3.0	68.7	2.74×10^{-13}	2.32	705	9.62
4.8 (run#2)	8.2	18.9	1.00×10^{-12}	0.635	495	2.63

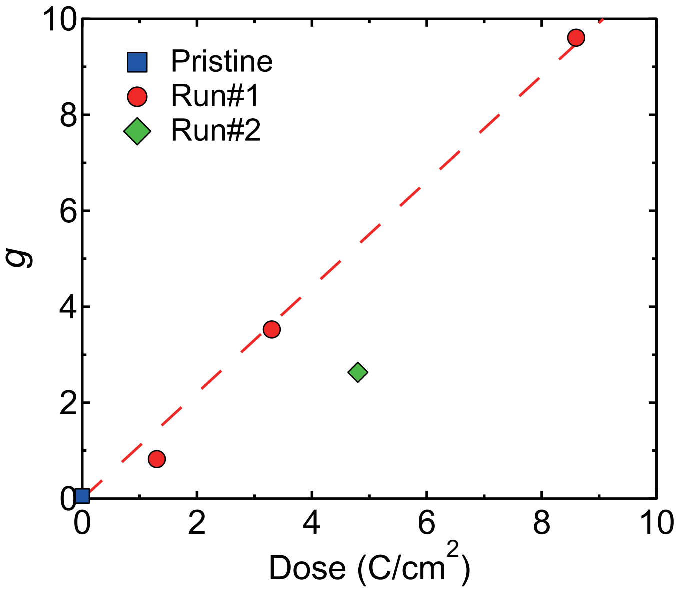

Fig. S4. Pair breaking parameter vs irradiation dose. The pair breaking parameter g as a function of irradiation dose. Dotted line is a guide for the eyes.

where ρ_0 is the residual resistivity for the irradiated samples and $\lambda_{\rm L}(0)$ is the London penetration depth at 0 K. Here, we assume that $\lambda_{\rm L}(0)$ is equal to $\lambda(0) = 387\,{\rm nm}$, which has been estimated in the previous penetration depth study for pristine samples [S7]. Thus, the single-band form (Eq. 4) can be applied to the 8.6 C/cm^2 irradiated sample. As a result, we obtained $\lambda(0) = 705\,{\rm nm}$ for the 8.6 C/cm^2 irradiated sample. For simplicity, we also assume the single-band form (Eq. 4) for the 1.3 and 3.3 C/cm^2 irradiated samples to obtain $\lambda(0)$. The obtained values of $\lambda(0)$ for the intermediate doses almost follow the linear increase in $\lambda(0)$ with irradiation dose (Fig. 1h), which we find reasonable in the first approximation. Note that the detailed choice of $\lambda(0)$ does not affect the conclusions on the s-wave superconductivity and the irradiation-induced change from anisotropic to isotropic gaps in CsV_3Sb_5.

We also estimated the pair-breaking parameter $g = \hbar/(\tau k_{\rm B} T_{\rm c0})$ at ambient pressure using τ in Table S1. The obtained parameters are summarized in Table S1. Note that the pair-breaking parameter g of the 4.8 C/cm^2 irradiated sample in run#2 deviates from a trend obtained in the samples irradiated in run#1 (Fig. S4). The value of the residual resistivity ratio (RRR) of the 4.8 C/cm^2 irradiated sample is located between those of the 1.3 and 3.3 C/cm^2 samples (Table S1), indicating that the amount of defects in the 4.8 C/cm^2

irradiated sample is between them.

We also estimated the pair-breaking parameter $g = \hbar/(\tau k_B T_{c0})$ at P_2 (which corresponds to the second peak pressure of the double superconducting dome) for each dose (Fig. S5), where $\tau = \mu_0 \lambda_L^2(0)/\rho_0$ at P_2 was calculated using the results of μSR measurements under pressure [S8]. Our results display that the suppression of T_c in the second superconducting phase is slower than that of the d-wave case with a sign-changing order parameter; it is rather close to the trend in MgB_2, which is a typical multiband s-wave superconductor with no sign change. These results support that CsV_3Sb_5 is an s-wave superconductor with no sign change both at ambient and high pressure.

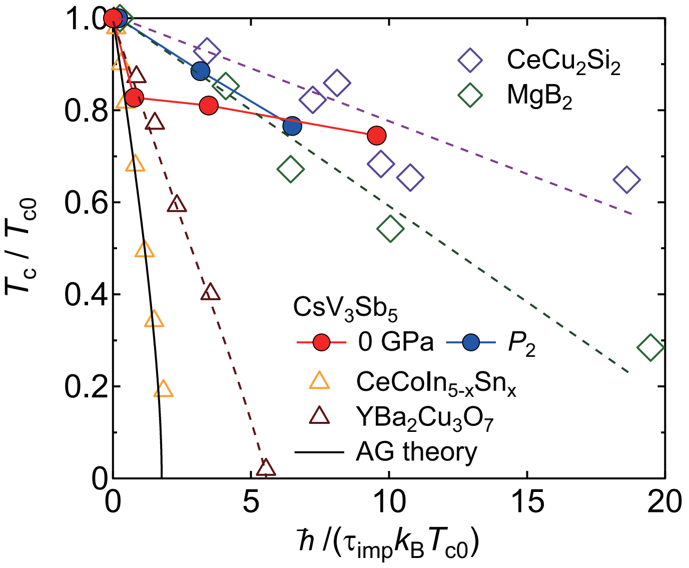

Fig. S5. Suppression of T_c in CsV_3Sb_5 at P_2 as a function of pair breaking parameter. For comparison, the same data as in Fig. 3d are shown.

V. FITTING ANALYSIS OF SUPERFLUID DENSITY

To analyze the overall temperature dependence of the normalized superfluid density $\rho_{\rm s}$, we used the so-called α model described by two independent gaps [S9],

$$\rho_{\rm s}(T) = x\rho_{\rm s,1}(T) + (1-x)\rho_{\rm s,2}(T), \quad (6)$$

where $\rho_{{\rm s},i}$ is the superfluid density on each band, and x is the weight of each band. In the previous study [S7], an isotropic two-gap s-wave model was used for the analysis of the superfluid density in CsV_3Sb_5. Therefore, we tried to fit the data with x as a free parameter and obtained a good fitting result with $x = 0.79$, which is consistent with the previous study [S7]. Then, we performed the same fitting analysis for the irradiated samples with $x = 0.79$. The dose dependence of the two gap values, Δ_1 and Δ_2, obtained from the fitting analysis is shown in Fig. S6. As the irradiation dose increases, Δ_1 and Δ_2 approach a value close to each other, but their averaged gap value $\Delta_{\rm ave}(= x\Delta_1 + (1-x)\Delta_2)$ decreases once and then increases, even though $T_{\rm c}$ decreases significantly. Such behaviour is physically unlikely, which probably comes from the assumption of the isotropic two-band model that ignores

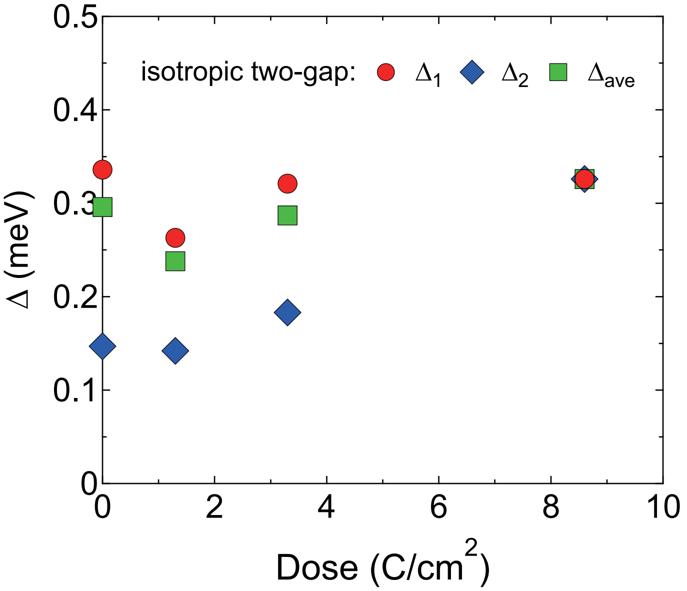

Fig. S6. Gap sizes obtained from the fitting analysis of superfluid density with the isotropic two-gap ***s*****-wave model as a function of dose.** Red circles and blue diamonds represent the gap values Δ_1 and Δ_2, respectively. Green squares represent the averaged gap value $\Delta_{\rm ave}$.

the anisotropy of the superconducting gap.

Thus, we next tried to fit the data with an anisotropic and isotropic two-gap s-wave model. Previous STS measurements in CsV_3Sb_5 [S10] have reported V-shaped $\mathrm{d}I/\mathrm{d}V$ spectra in the superconducting state, which implies the existence of an anisotropic superconducting gap. In addition, recent theoretical calculations suggest the emergence of anisotropic s-wave superconductivity mediated by bond-order fluctuations [S11]. Thus, we adopted the two-gap s-wave model with anisotropic and isotropic gaps. In this model, $\rho_{s,1}$ ($\rho_{s,2}$) corresponds to the superfluid density on the anisotropic s-wave gap with 6-fold symmetry (isotropic s-wave gap) (see Fig. 3b). We fixed the value of x under the assumption that the Fermi surfaces (FSs) derived from the V d-orbitals are responsible for the anisotropic s-wave gap, whereas the FS from the Sb p-orbitals contributes to the isotropic s-wave gap. The value of x is determined by the following equation:

$$x = \frac{n_1/m_1^*}{n_1/m_1^* + n_2/m_2^*}, \tag{7}$$

where n_i and m_i^* are the carrier density and effective mass of the i-th band, respectively. We estimated n_i/m_i^* from the band structure calculations in Ref. [S6], and obtained $x \approx 0.6$.

The anisotropic s-wave gap with 6-fold symmetry and isotropic s-wave gap can be given by

$$\Delta_1(T,\phi) = \Delta_1(T)\Omega(\phi) = \Delta_1(T)\frac{1+\alpha\cos(6\phi)}{\sqrt{1+\alpha^2/2}}, \tag{8}$$

$$\Delta_2(T,\phi) = \Delta_2(T), \tag{9}$$

respectively, where α is the anisotropic parameter, $\Delta_i(T)$ is the temperature-dependent part, and $\Omega(\phi)$ is the angular-dependent part (which is normalized on the FS to satisfy the relation

TABLE S2. **Superconducting gap values of pristine and irradiated CsV_3Sb_5 obtained from fitting analysis.**

Dose (C/cm^2)	T_c^{TDO}(K)	α	Δ_1(meV)	$\Delta_{1,\mathrm{max}}$(meV)	$\Delta_{1,\mathrm{min}}$(meV)	Δ_2(meV)
pristine	2.89	0.626	0.345	0.513	0.118	0.315
1.3	2.2	0.488	0.286	0.402	0.138	0.227
3.3	2.15	0.291	0.308	0.389	0.214	0.287
8.6	2.05	0.0005	0.312	0.311	0.312	0.336

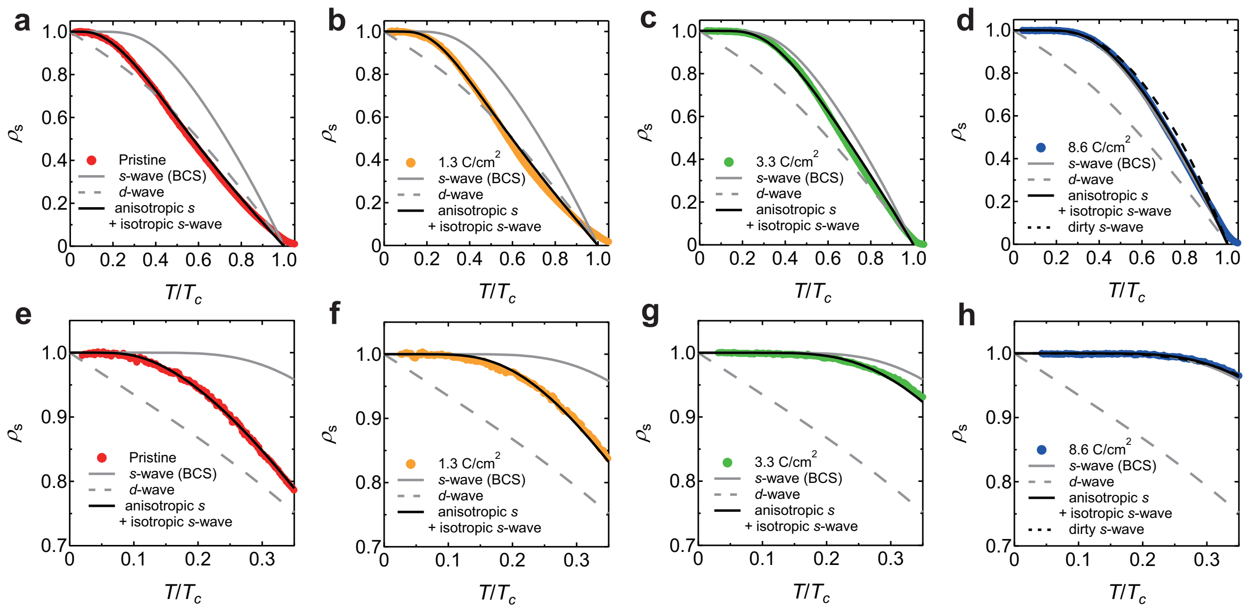

Fig. S7. **Fitting analysis of the temperature dependence of superfluid density in pristine and irradiated $\mathbf{CsV_3Sb_5}$. a-d**, Normalized superfluid density $\rho_{\rm s}$ as a function of the reduced temperature $T/T_{\rm c}$. Solid circles represent the experimental data for the pristine (**a**), 1.3 (**b**), 3.3 (**c**), and 8.6 (**d**) C/cm^2 irradiated samples. Black solid lines are the fitting curves of the multi-gap model (clean limit). Black dashed line in **d**,**h** is the fitting curve of the single-gap model (dirty limit). Gray solid and dashed lines are the calculated temperature dependences of $\rho_{\rm s}(T)$ in the conventional s-wave (clean limit) and unconventional d-wave (clean limit) cases, respectively. **e-h**, Enlarged view of the low-temperature region in **a-d**.

$\langle\Omega(\phi)\rangle_{\rm FS} = 1$). We solved the gap equation to obtain $\Delta_i(T)$. As a result of these analyses, we find that $\rho_s(T)$ can be well fitted to the above two-gap model for the pristine and 1.3, 3.3, and 8.6 C/cm^2 irradiated samples (Fig. S7). The gap values obtained from the fitting with the multi-gap model are summarized in Table S2. For the 8.6 C/cm^2 irradiated sample with the relatively large $\xi/l = 2.32$ (see Table S1), the $\rho_s(T)$ curve approaches the single-gap model with $\Delta(0) = 1.65k_{\rm B}T_{\rm c}$ in the dirty limit described by the following equation [S12]:

$$\rho_s^{\rm dirty}(T) = \frac{\Delta(T)}{\Delta(0)}\tanh\frac{\Delta(0)}{2k_{\rm B}T}, \tag{10}$$

where $k_{\rm B}$ is the Boltzmann constant (see Fig. S7d,h).

Supplementary References

[S1] Bois, P. Etude des défauts ponctuels dans le bismuth. *CEA Report* No. R–5389 (1987). URL `https://inis.iaea.org/collection/NCLCollectionStore/_Public/18/082/18082185.pdf`.

[S2] Ortiz, B. R. *et al.* New kagome prototype materials: discovery of KV_3Sb_5, RbV_3Sb_5, and CsV_3Sb_5. *Phys. Rev. Materials* **3**, 094407 (2019). URL `https://link.aps.org/doi/10.1103/PhysRevMaterials.3.094407`.

[S3] Yu, F. H. *et al.* Concurrence of anomalous Hall effect and charge density wave in a superconducting topological kagome metal. *Phys. Rev. B* **104**, L041103 (2021). URL `https://link.aps.org/doi/10.1103/PhysRevB.104.L041103`.

[S4] Li, Y. *et al.* Tuning the competition between superconductivity and charge order in the kagome superconductor $Cs(V_{1-x}Nb_x)_3Sb_5$. *Phys. Rev. B* **105**, L180507 (2022). URL `https://link.aps.org/doi/10.1103/PhysRevB.105.L180507`.

[S5] Yang, S.-Y. *et al.* Giant, unconventional anomalous Hall effect in the metallic frustrated magnet candidate, KV_3Sb_5. *Science Advances* **6**, eabb6003 (2020). URL `https://www.science.org/doi/abs/10.1126/sciadv.abb6003`.

[S6] Ortiz, B. R. *et al.* Fermi surface mapping and the nature of charge-density-wave order in the kagome superconductor CsV_3Sb_5. *Phys. Rev. X* **11**, 041030 (2021). URL `https://link.aps.org/doi/10.1103/PhysRevX.11.041030`.

[S7] Duan, W. *et al.* Nodeless superconductivity in the kagome metal CsV_3Sb_5. *Science China Physics, Mechanics & Astronomy* **64**, 1–6 (2021). URL `https://doi.org/10.1007/s11433-021-1747-7`.

[S8] Gupta, R. *et al.* Two types of charge order in the superconducting kagome material CsV_3Sb_5. *arXiv preprint arXiv:2203.05055* (2022). URL `https://arxiv.org/abs/2203.05055`.

[S9] Kogan, V. G., Martin, C. & Prozorov, R. Superfluid density and specific heat within a self-consistent scheme for a two-band superconductor. *Phys. Rev. B* **80**, 014507 (2009). URL `https://link.aps.org/doi/10.1103/PhysRevB.80.014507`.

[S10] Xu, H.-S. *et al.* Multiband superconductivity with sign-preserving order parameter in kagome

superconductor CsV_3Sb_5. *Phys. Rev. Lett.* **127**, 187004 (2021). URL https://link.aps.org/doi/10.1103/PhysRevLett.127.187004.

[S11] Tazai, R., Yamakawa, Y., Onari, S. & Kontani, H. Mechanism of exotic density-wave and beyond-Migdal unconventional superconductivity in kagome metal AV_3Sb_5 (A = K, Rb, Cs). *Science Advances* **8**, eabl4108 (2022). URL https://www.science.org/doi/abs/10.1126/sciadv.abl4108.

[S12] Tinkham, M. Introduction to Superconductivity (McGraw-Hill, 1996).
